# Characterisation of the Putative Effector Interaction Site of the Regulatory HbpR Protein from *Pseudomonas azelaica* by Site-Directed Mutagenesis

**DOI:** 10.1371/journal.pone.0016539

**Published:** 2011-02-17

**Authors:** Christelle Vogne, Hansi Bisht, Sagrario Arias, Sofia Fraile, Rup Lal, Jan Roelof van der Meer

**Affiliations:** 1 Department of Fundamental Microbiology, University of Lausanne, Lausanne, Switzerland; 2 Department of Zoology, University of Delhi, Delhi, India; 3 National Centre for Biotechnology, CSIC, Madrid, Spain; University of Massachusetts Medical School, United States of America

## Abstract

Bacterial transcription activators of the XylR/DmpR subfamily exert their expression control via σ^54^-dependent RNA polymerase upon stimulation by a chemical effector, typically an aromatic compound. Where the chemical effector interacts with the transcription regulator protein to achieve activation is still largely unknown. Here we focus on the HbpR protein from *Pseudomonas azelaica*, which is a member of the XylR/DmpR subfamily and responds to biaromatic effectors such as 2-hydroxybiphenyl. We use protein structure modeling to predict folding of the effector recognition domain of HbpR and molecular docking to identify the region where 2-hydroxybiphenyl may interact with HbpR. A large number of site-directed HbpR mutants of residues in- and outside the predicted interaction area was created and their potential to induce reporter gene expression in *Escherichia coli* from the cognate P*_C_* promoter upon activation with 2-hydroxybiphenyl was studied. Mutant proteins were purified to study their conformation. Critical residues for effector stimulation indeed grouped near the predicted area, some of which are conserved among XylR/DmpR subfamily members in spite of displaying different effector specificities. This suggests that they are important for the process of effector activation, but not necessarily for effector specificity recognition.

## Introduction

Transcription activators of the XylR/DmpR subfamily of σ^54^-dependent regulatory proteins play pivotal roles in controlling gene expression in bacterial aromatic compound catabolism [1], [2]. Classical and very well characterized examples include XylR, the primary activator for the *xyl* genes of the TOL plasmid for toluene and xylene degradation in *Pseudomonas putida* mt-2 [3], DmpR, the sole transcription activator of the *dmp* genes for phenol and *o*-cresol metabolism in *Pseudomonas* sp. strain CF600 [4], and TouR, from *Pseudomonas stutzeri* OX1 [5]. A large number of more diverse members of the same subfamily have been identified in the course of the recent years, among which are PhnR from *Burkholderia sartisoli* RP007 (regulating phenanthrene metabolism) [6], HbpR from ‘*Pseudomonas azelaica*’ (activating 2-hydroxybiphenyl metabolism) [7] and TbuT from *Burkholderia pickettii* PKO1 [8].

XylR/DmpR subfamily members belong to the even larger class of NtrC-type transcription regulators, which are involved in a variety of physiological processes in response to diverse environmental signals [9]. Generally, these transcriptional activators act at a distance of 100 to 200 bp from the actual promoter by binding to what are called enhancer-like elements or upstream activating sequences (UAS) [10]. In addition, they specifically interact with σ^54^ RNA polymerase [1]. A further hallmark of proteins from this family is the presence of two conserved domains, one of which is called the central C-domain and contains a triple-AAA ATPase motif [11]. The C-domain is supposed to interact with σ^54^ RNA polymerase and hydrolyzes ATP, perhaps to facilitate open transcriptional complex formation. The second conserved feature of these proteins is a carboxy terminal D-domain, which contains a typical helix-turn-helix DNA binding motif and is implicated in interaction with the UAS-DNA [12]. In contrast to NtrC, members of the XylR/DmpR subfamily have a distinct N-terminal or A-domain necessary for recognition of chemical effector molecules that unleashes activity of the transcription activator [13]. A further small region called the B-domain or Q-linker because of its abundance in glutamine residues, connects the A- and the C-domain. It is supposed to act as a flexible molecular hinge, releasing intramolecular repression by the A-domain and exposing the ATP-ase activity of the C-domain upon recognition of the effector [14]. Indeed, XylR and DmpR mutants devoid of their A-domain act as constitutive transcription activators on their cognate promoters [15], [16]. Importantly, however, an A-domain deletion of the distantly related HbpR protein (see below) is constitutively repressed [17].

Despite extensive genetic and biochemical data on XylR and DmpR, there is still no clear picture on the A-domain residues implicated in effector interaction, neither does a clear hypothesis exists on the mechanism of effector-mediated triggering of the activation process. Most information so far comes from the analysis of XylR and DmpR, from screening of spontaneous mutants [18], of mutants obtained by directed evolution [19] or by DNA family shuffling [13], [20], and of mutants obtained by site-directed mutagenesis [21]. Attempts to obtain direct structural information on the proteins of this subclass have been frustrated by the difficulty to purify and stabilize the full protein. Nevertheless, a structural model for the XylR A-domain was proposed based on low but pertinent similarity to the A-chain of eukaryotic catechol O-methyl transferase [22]. This model, however, consists of only a single XylR A-domain protomer whereas the current hypothesis predicts that proteins from this class undergo an activation cycle of multimerization and multimer disassembly [23].

**Figure 1:**
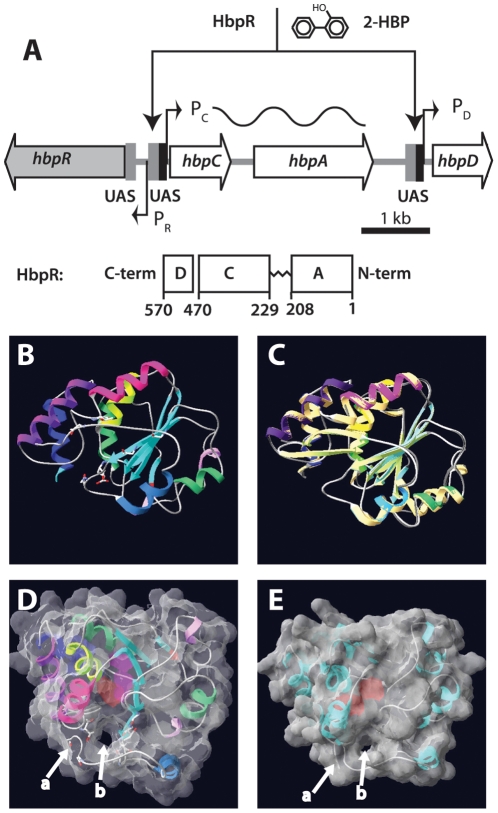
Overview of the *hbp* regulatory system and tertiary structure modeling of the HbpR A-domain using a previously established XylR model as template. (A) Organization of the *hbp* genes and the location of the HbpR binding sites (UAS, upstream activating sequences) in front of the P_C_ and P_D_ promoters. HbpR domains are depicted to scale according to the predictions by Jaspers et al [7]. (B) to (E) Fitting used swiss-model and was performed on XylR A-domain PDB coordinates as calculated by Devos et al [22]. (B) Ribbon model of HbpR A-domain residues 11–209, with predicted coils, alpha-helices and beta-sheets indicated. (C) Superposition of the predicted HbpR and XylR A-domains in the same configuration as A. (D) Tertiary structure model of HbpR A-domain with calculated molecular surface at 1.4Å and 40% transparency, in order to see the helical, coil and sheets. Model turned into a position which enables visualization of the proposed tunnel entry (b). C-terminal end of coil ending the A-domain indicated with an arrow at (a). Pinkish region in the centre of the A-domain illustrates a predicted cavity within the A-domain. (E), as C but now for the XylR A-domain, with exception of the ten most C-terminal residues, which otherwise are predicted to occlude the tunnel.

The goal of the current work was to identify the residues critical for effector-mediated triggering in the HbpR protein from *P. azelaica* [7], [17]. In its native host, the *hbpR* gene product regulates expression from two promoters, called the P*_C_* and P*_D_* promoters, which are located in front of two small operons (*hbpCA* and *hbpD*) encoding the enzymes for initial steps of 2-hydroxybiphenyl (2-HBP) degradation (Fig. 1A) [24]. The *hbpR* gene is located directly upstream of and is divergently oriented from the *hbpCA* genes. HbpR displays only 37% amino acid sequence identity with XylR, and in contrast to XylR and DmpR, is responsive to biaromatic compounds such as 2-HBP, 2,2′-dihydroxybiphenyl, 2-aminobiphenyl and 2-hydroxydiphenylmethane [7]. XylR and HbpR display detectable but little crossbinding to each other's DNA binding sites although hybrid promoters can be produced that are activated by both XylR and HbpR in the same cell [25]. In contrast to XylR and DmpR, the Q-linker of HbpR is shorter and A-domain deletions of HbpR result in a constitutive repressor protein [17]. Since such A-domain deletions are made without any protein structure basis, it is possible that they accidentally produce different effects in HbpR and XylR or DmpR.

In order to decipher possible determinants in the A-domain of HbpR for 2-HBP-mediated triggering, we assumed that 2-HBP would interact with specific residues exposed to the A-domain surface. To make a more rational guess on the choice of residues to investigate, we expanded the modeling approach previously developed for the A-domain of XylR [22] to predict a tertiary structure for that of HbpR, and predicted the regions of possible 2-HBP interaction using small ligand docking approaches. Amino acid residues in the predicted effector-interaction region and in control regions outside were changed by site-directed mutagenesis. The integrity and activation potential of the resulting mutant proteins by the natural effector 2-HBP and the two non-natural effectors toluene and 2-chlorobiphenyl was tested in an *Escherichia coli* based heterologous expression system. Our results essentially confirm the modeling hypothesis and expand our understanding of several critical residues in not only in HbpR but in XylR and DmpR for effector interaction.

## Results

### Prediction of A-domain folding and the 2-HBP interaction site on HbpR

To make a rational prediction of which amino acids in the HbpR A-domain could be implied in effector interaction, its tertiary structure was modeled. Because no crystal structure of the effector binding domain of HbpR or close relative has been determined, the domain was modeled using a bioinformatics approach similar as proposed earlier for XylR [22]. Directly fitting a tertiary structure model for the HbpR A-domain failed because of too low homology to any existing structures in the PDB database [26]. The first 218 amino acids of HbpR were thus structurally aligned to the XylR and DmpR A-domain computational models [22] as templates using the program swiss-model [26], [27]. The computed HbpR A-domain model for the amino acids 9-211 displayed eight alpha helices and five beta strands (Fig. 1B, C). As expected using these templates, the predicted shape for the HbpR A-domain was highly similar to those of XylR and DmpR with exception of a few loops (Fig. 1D, E). The HbpR A-domain C-terminal end is predicted to be coiled instead of forming beta sheets as in XylR and DmpR, but it should be noted that the model does not take the A- and C-domain connection of the protein into account. Interestingly, the HbpR A-domain model predicted one face of the tertiary structure to have an overall more negative electric potential than the opposite face, which may favor dimeric A-domain interactions (not shown).

**Figure 2:**
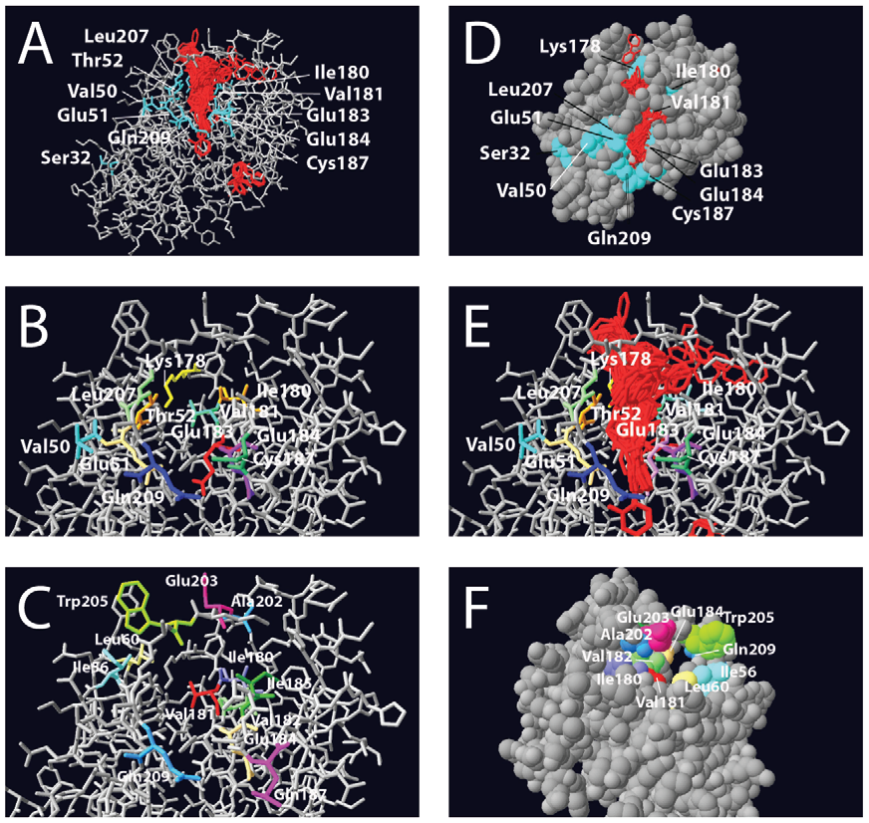
Details of the modeled tertiary structure of the HbpR A-domain, showing amino acid residues that were mutated in this study and the region onto which 2-HBP is predicted to be bound. (A) Results of 1000 iterations of 2-HBP (in red) docking calculations using gramm onto the predicted HbpR A-domain protein surface, whilst indicating the position of residues altered to Phe. (B) Close-up of the same, but without the docked 2-HBP positions. (C) as for B, now highlighting the other changed residues. (D) Van der Waals-filled model slightly turned compared to A, in order to indicate the region of 2-HBP docked molecules. (E), as B, but with 2-HBP docked positions. (F) Turned van der Waals-filled model showing the tunnel from the other side of the entry.

The A-domain model for HbpR was then used as a template to predict the possible sites of interaction with its effector 2-HBP (Fig. 2A, D, E). Potential sites for 2-HBP interaction were calculated by using the program gramm, which uses Fast Fourier transformation to predict the energetically most favorable matches of a ligand on the modeled protein surface [28]. Interestingly, gramm calculations predicted that there would be an ‘interface’ region most favorable for interaction with 2-HBP rather than a single residue or active site, which upon closer inspection of the model seemed to provide a cavity (Fig. 2A). Among one thousand iterations, the program predicted almost exclusively interactions in this particular region. A number of amino acid residues such as E184 were located in this region (Fig. 2A, B), which upon mutation in XylR had been demonstrated to broaden effector-mediated induction [29]. In addition, a similar region had been predicted from the XylR A-domain model to be of potential interest to effector binding, even though few mutations had been generated in that part of the protein [22]. The main hypothesis in this work was therefore that this interface region would be critical for 2-HBP-mediated triggering of HbpR activation.

### Design and construction of HbpR mutants

Two groups of mutations were created to validate or refute our hypothesis: a first group, which concentrated on a number of amino acid residues in this region conserved between HbpR, XylR and DmpR. Mutations in this group were designed to alter the chemical nature of the residue (i.e., charged to non-charged, hydrophobic to hydrophilic). In the second group we designed mutations, which would ‘block’ the cavity by the bulky amino acid phenylalanine. Because such drastic replacements by Phe could have secondary effects on protein performance, we created a number of control mutations on residues not predicted to be directly at the cavity interface (Fig. 2).

**Figure 3:**
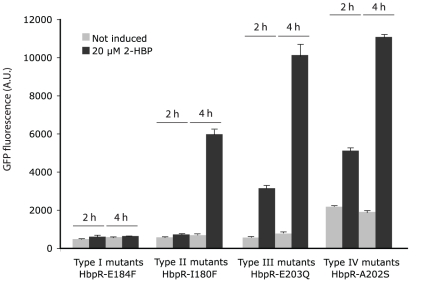
Exemplary effects of HbpR A-domain mutations on inducible expression. Measured fluorescence intensities of *Escherichia coli* cells carrying a plasmid with a promoterless *egfp* under control of the HbpR-dependent P*_C_*-promoter in the presence or absence of 20 µM 2-HBP as inducer. EGFP expression was measured on whole cells at two time points and corrected for culture turbidity. Type I to IV correspond to differently shaded entries in Table 1. Note the delayed response in Type II mutants and the higher background in the absence of inducer in type IV mutants.

**Figure 4:**
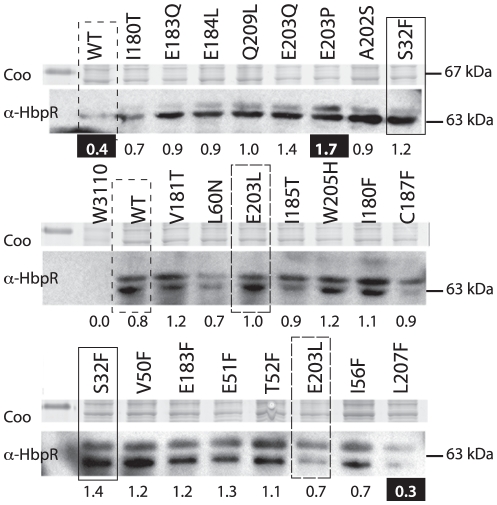
HbpR (mutant) expression in *E. coli* from pHBP269A0-plasmids, i.e., those which were used for 2-HBP induced EGFP expression from P*_C_*. Coo, Coomassie-Blue-stained SDS-PAGE gel fragment around 67 kDa. α-HbpR, bands on gel detected in Western blotting with anti-HbpR antibodies. Relevant mutations are indicated; note that wild-type and several mutants were analyzed twice. Numbers below the gel fragments indicate the average normalized intensities of both HbpR bands for each mutant or wild-type. Those numbers highlighted in white on black background point to values below or above the 25 and 75% quantiles of all measured intensities.

An overview of all mutants constructed in the first and the second group is presented in Table 1. All mutations in the HbpR A-domain were constructed by PCR with mutated oligonucleotides and verified by DNA sequencing. Subsequently, the mutated A-domain sequences were used to replace the gene region for the native A-domain in *hbpR* on an expression vector in *E. coli*, with which we could test 2-HBP inducible *egfp* expression under control of the HbpR-dependent P*_C_* promoter. This expression vector results in the addition of a His6-tag to the N-terminal end of the protein. All mutants were tested in *E. coli* for EGFP expression during exponential growth in the presence or absence of 20 µM 2-HBP, which is the cognate effector for the HbpR-P*_C_* system. Table 1 gives representative EGFP induction values after 2 and 4 h induction time compared to those of the strain carrying wild-type HbpR. In general and for all mutants, we observed four types of effects: (i) complete loss of activation with respect to the wild-type (type I), (ii) two-fold loss of induction potential in 2 h but not 4 h incubation periods (type II), (iii) no effect compared to the wild-type (type III), and (iv) considerable increase of background expression (type IV, Table 1, Fig. 3). Protein extracts of the same strains were analyzed by Western blotting using an M13-V_HH_ camel antibody to verify (mutant) HbpR expression (Fig. 4). Surprisingly, all Westerns showed two bands, which likely correspond to His6-tagged HbpR (or mutant, 64.1 kDa) and HbpR (mutant) without His6-tag (62.8 kDa). The reason for the production of two N-terminally different HbpR proteins probably lies in the use of an alternative start codon further downstream. The expression level of most HbpR mutant proteins in *E. coli* was similar to the wild-type, except for L207F (low outlier) and E203P (high outlier) (Fig. 4; Text S1).

**Table 1:** Fluorescence intensities in *Escherichia coli* expressing EGFP from P*_C_* under control of HbpR wild-type or its mutants, in the presence or absence of 2-hydroxybiphenyl.

Mutant Class	Residue	2 h induction time				4 h induction time			
		NI^a^	Ratio to WT	Induced	IF	NI	Ratio to WT	Induced	IF
I	Cys187Phe	505±18^b^	0.96	582±53	1.2	538±14	0.97	597±18	1.1
I	Glu184Phe	487±21	0.92	615±92	1.3	589±23	1.06	648±13	1.1
I	Glu184Leu	536±11	1.01	657±43	1.2	603±43	1.08	648±13	1.1
I	Thr52Phe	544±21	1.03	665±14	1.2	639±24	1.15	1411±72	2.2
II	Ile180Phe	577±12	1.09	723±21	1.3	688±22	1.24	5993±253	8.7
II	Val182Thr	537±18	1.02	878±84	1.6	572±23	1.03	6513±35	11.4
II	Ile180Thr	458±17	0.86	1709±48	3.7	474±17	0.85	3854±100	8.1
II	Leu207Phe	482±18	0.91	1213±53	2.5	542±25	0.97	5733±144	10.6
II	Ile56Thr	481±16	0.91	2161±167	4.5	582±16	1.05	6550±139	11.3
III	Glu203Leu	544±18	1.03	2731±80	5.0	662±21	1.19	8571±146	12.9
III	Gln209Leu	546±21	1.03	2977±127	5.5	697±32	1.25	8331±162	12.0
III	Glu42Phe	472±11	0.89	2792±225	5.9	497±27	0.89	7853±125	15.8
III	Glu203Gln	569±14	1.08	3152±147	5.5	751±29	1.35	10141±650	13.5
III	Glu203Pro	580±14	1.1	3147±178	5.4	754±34	1.35	11120±530	14.7
III	Val50Phe	493±12	0.93	3151±104	6.4	574±29	1.03	8028±91	14.0
III	Lys178Phe	510±11	0.96	3334±169	6.5	611±23	1.09	8567±145	14.0
III	**Wild-type**	529±21	1	3503±260	6.2	557±21	1	8858±195	15.9
III	Trp205His	540±19	1.02	3554±231	6.6	673±32	1.21	8931±263	13.3
III	Val181Thr	563±12	1.06	3589±136	6.4	690±41	1.24	9536±257	13.8
III	Leu60Asn	511±13	0.97	3615±103	7.1	618±16	1.11	9443±172	16.0
III	Glu183Phe	547±18	1.03	3653±136	6.5	683±38	1.23	9903±87	14.5
III	Glu183Gln	595±15	1.12	3836±125	6.5	730±29	1.31	9493±126	13.0
III	Gln188Glu	692±11	1.31	3853±146	5.6	839±42	1.51	10766±118	12.8
III	Ile185Thr	470±16	0.89	3896±163	8.3	548±21	0.98	8094±218	14.8
III	Gln209Phe	456±23	0.86	3950±165	8.7	612±18	1.09	7465±187	12.2
IV	Val181Phe	1049±26	1.98	4368±104	4.2	1647±63	2.96	10185±212	6.2
IV	Ser32Phe	828±19	1.56	4626±108	5.6	1598±72	2.87	11432±283	7.2
IV	Ala202Ser	2184±35	4.18	5156±153	2.4	1923±57	3.45	11072±157	5.8

a), NI, non induced conditions and ratio of NI-fluorescence in mutant and that of wild-type; IF, induction factor, calculated by dividing culture fluorescence with 2-hydroxybiphenyl by that of the culture in the absence of 2-hydroxybiphenyl.

b) Averages from biological triplicates with calculated standard deviation.

The first group of mutations directed to changing the chemical character of conserved residues among XylR/DmpR/HbpR A-domains produced the following results. E184L (equivalent position in XylR E172, Text S1) completely abolished EGFP expression from the HbpR dependent P*_C_* promoter (type I). To a lesser extent, also mutations V182T and I180T drastically reduced EGFP induction upon 2-HBP addition – mostly after 2 h, but after 4 h induction time the difference to the wild-type was less pronounced (type II, Table 1, Fig. 3). Other residues, mutation of which reduced activation potential by 2-HBP, were I56T, E203L, E203Q and E209L (Table 1). By contrast, mutation of the chemical character of residues in this vicinity, e.g., W205H, V181T, L60N, Q188E and I185T, did not significantly affect 2-HBP-dependent activation in *E. coli* (type III). Interestingly, changing Ala202 to Ser resulted in a fourfold higher EGFP expression in the absence of 2-HBP as compared to wild-type HbpR (type IV). This suggested that several residues in this area indeed affected activation of expression by 2-HBP, but only Glu184 seemed absolutely critical.

Next, we created Phe-substitutions in a number of residues in the predicted interface area, which we suspected could ‘block’ a cavity seen on the surface of the model. As controls, a number of randomly chosen other residues were also substituted by Phe (Fig. 2D). The effect of those mutations was again analyzed by HbpR-dependent 2-HBP-inducible EGFP expression in *E. coli* (Table 1). As for mutant E184L, also E184F completely abolished inducible *egfp* expression from P*_C_*. Similar effects were caused by mutations C187F and T52F. Phenylalanine substitutions in Ile180 and Leu207 resulted in the delayed induction phenotype (type II). Mutations in the majority of residues had basically no effect on the magnitude or kinetic induction with 2-HBP (Glu51, Val50, Lys178, Glu183 and Gln209). All of these were located more or less in the vicinity of the proposed cavity (Fig. 2C, D), but not as close to the 2-HBP interaction region as, e.g., Thr52, Ile180, Val182 or Glu184. Interestingly, two Phe substitions (at Ser32 and Val181) produced HbpR-mutants with higher background expression in the absence of 2-HBP (type IV, Table 1, Fig. 3).

For a number of residues multiple substitutions were created, which almost in all cases produced the same effect. Both mutations in Glu184 (to Phe or to Leu), abolished induction with 2-HBP, and also both mutations in Ile180 (to Phe or to Thr) decreased 2-HBP induction (Table 1). All mutations created in Glu203 (to Leu, Gln and Pro) were more or less without large effect on 2-HBP induction. Also both mutations in Glu183 (Phe and Gln) produced the same effect. On the contrary, Val181Thr had no effect, but Val181Phe produced a higher non-inducible background. The same was found for Gln209, for which a change to Leu reduced, but change to Phe slightly increased the magnitude of *egfp* induction with 2-HBP.

### HbpR mutant integrity

**Figure 5:**
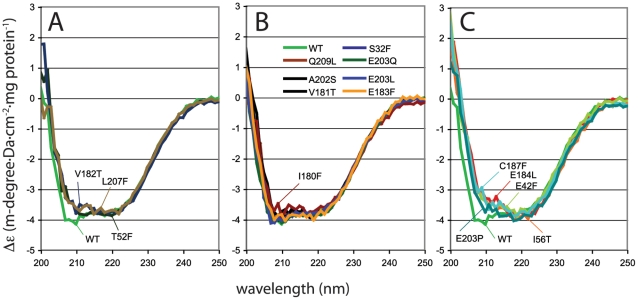
Circular dichroism spectra of purified His6-tagged HbpR wild-type protein or of sixteen purified HbpR A-domain mutants, between 200 and 250 nm, at a protein concentration of ≈0.3 mg/ml. Spectra were normalized to Δε, as indicated in the Experimental Procedures section, and grouped to reveal similar dichroism trends. (A) HbpR wild-type and mutants V182T, L207F and T52F (type II effects with delayed and lower induction by 2-HBP). (B) Mutants with similar dichroisms as HbpR wild-type. (C) Mutants with the most strong aberration of the wild-type circular dichroism trace, of which C187F and E184L completely abolished activation by 2-HBP, but E42F and E203P having no major effect on 2-HBP dependent induction in *E. coli*.

Western blotting with an anti-HbpR M13-displayed V_HH_ camel antibody suggested (within the accuracy of this technique) that most HbpR mutant proteins were produced to the same level in *E. coli* (Fig. 4), except for L207F (lower than expected) and E203P (higher than expected). This indicated that differential EGFP expression in *E. coli* carrying a mutant *hbpR* gene was not due to complete misfolding or degradation of the protein, but rather due to a critical amino acid change in the effector binding region. In particular E184L, I180T, I180F, T52F and C187F, which were the mutations causing the largest decrease of 2-HBP-dependent EGFP expression from P*_C_*, resulted in HbpR mutant proteins that were expressed in *E. coli* within the normal range observed for all (Fig. 4). To corroborate this further, we purified a number of (mutant) HbpR proteins and compared their circular dichroism spectra between 200 and 250 nm. Sixteen mutant HbpR proteins and HbpR wild-type (all tagged with His6) were hereto purified by Ni-NTA chromatography, dialysed and diluted to 0.3 mg protein per ml (Fig. 5). For reasons of protein stability, it was not possible to completely omit traces of EDTA and glycerol from the dialysis buffer. As a result no reliable spectra below 198 nm could be recorded (not shown).

Whereas identical spectral traces are usually interpreted as proteins having the same solution conformation, the HbpR wild-type and 15 mutant proteins produced similar but not identical traces. Broadly we detected three types of spectra in the region between 205 and 240 nm (Fig. 5). Most mutant proteins differed very little from the HbpR wild-type circular dichroism (Fig. 5B). Three mutants (V182T, L207F and T52F) deviated specifically in the region 206–212 nm (Fig. 5A), and mutants C187F, E184L, E42F, E203P and I56T differed more strongly in the region 205–220 nm (Fig. 5C). This indicates, therefore, that some HbpR mutant proteins adopt different configurations than HbpR wild-type (folding, or multimerization in solution). However, since mutant and wild-type protein expression in *E. coli* was more or less similar (except for L207F and E203P), we conclude that different circular dichroism profiles reflect the immediate refolding effect of a mutation but are not indicative for complete misfolding, or else the phage antibody would not have recognized the protein. Moreover, a number of mutant HbpR proteins with slightly different scans still retained normal induction potential. For example, E203P and E42F showed circular dichroism scans clearly different from wild-type HbpR and similar to C187F and I56T. Yet, E203P and E42F maintained induction potential similar as wild-type HbpR, whereas C187F and I56T were impaired (Table 1). By contrast, proteins V182T, L207F and T52F were all impaired in activation potential and their circular dichroisms differed from wild-type. Therefore, we conclude that some mutations cause different partial folding, but this does not necessarily lead to an overall change in protein configuration such that it renders the protein inactive and would cause the lack of induction with 2-HBP. Thus, effects on 2-HBP-dependent EGFP expression from P*_C_* must have been the genuine consequence of a change in a critical effector binding region or residue.

### Complementation of the mutants with the wild type HbpR

**Figure 6:**
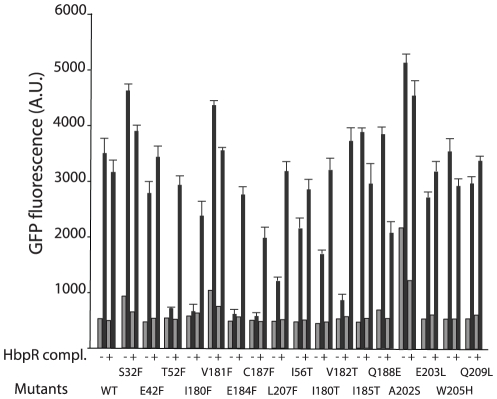
EGFP expression from the HbpR-dependent P*_C_*-promoter in *E. coli* in the presence (black bars) or absence (grey bars) of 2-HBP as inducer measured for the different HbpR A-domain mutants after 2 h induction time, and either complemented with a second plasmid carrying wild-type HbpR (pHB240) or not. Note the partial ‘rescue’ of the abolished phenotype in T52F, I180F, E184F, C187F and V182T by wild-type HbpR, suggesting that the mutant proteins are not dominantly negative over the wild-type. Results indicate the mean of triplicate incubations, plus the calculated standard deviation.

Next, we tested whether the created HbpR mutations were dominant over wild-type HbpR, which would be a further indication for their activity in *E. coli*, since we previously demonstrated that an HbpR mutant devoid of the A-domain was dominant negative on wild-type HbpR [17]. Hereto, the A-domain mutant strains of HbpR in *E. coli* were complemented with a plasmid expressing wild-type *hbpR* from its native promoter (pHBP124). For all type I mutants (loss of induction), complementation with wild-type HbpR restored 2-HBP inducible activity although not to the level of wild type response (Fig. 6). Mutants C187F and I180F reverted to 3/4^th^ of the lost activity upon complementation with wild-type. This might be the result of formation of heteromultimers between wild-type HbpR and mutant protomers, which do not fully restore functionality. It is worth noticing that for mutations W205H, I185T and Q188E, which did not affect the activity of the protein, complementation reduced the response to 2-HBP. This effect could also be seen with the semi-constitutive mutants A202S, V181F and S32F; complementation with the wild-type HbpR decreased the response upon induction (Fig. 6). Such mutations may therefore affect heterodimer formation.

Finally, we tested whether any of the mutants were different in response to aromatic compounds not known to activate wild-type HbpR. Hereto we chose 2-chlorobiphenyl (at 100 µM), which is the effector for certain HbpR mutants obtained by directed evolution approaches [30], and toluene (at 20 µM), which is an effector for XylR. Incubation of the HbpR mutant series or wild-type in *E. coli* with 2-chlorobiphenyl did not produce any significant induction compared to non-amended cultures (not shown). None of the mutants or wild-type were responsive to toluene either. Toluene (at 20 µM) reduced by 10% the induction obtained with 32 µM 2-HBP in a co-induction assay for wild-type HbpR and the mutants I180F, V181T, I185T, E203L, E203Q and W205H (not shown). In a few other mutants (I56T, E183F and K178F) coincubation with 20 µM toluene and 32 µM 2-HBP enhanced EGFP expression by ≈20% compared to 32 µM 2-HBP alone. This indicates that the created mutations do not ‘widen’ the effector spectrum.

## Discussion

Although previous work by several groups have clearly demonstrated the importance of the A-domain of proteins of the XylR/DmpR subfamily of transcription activators in effector recognition, the actual effector ‘binding’ region and the type of effector interaction have largely remained elusive. A plethora of A-domain mutations has been produced for XylR and DmpR (Text S1), which highlighted several residues in activation function. A conceptual breakthrough was proposed by Devos and coworkers in 2002, who developed a structural model for the XylR (and DmpR) A-domains on the basis of a weak but significant structural homology to catechol O-demethylase from *Rattus norvegicus*. Placement of the various mutated residues and their effects on the modeled structure seemed to indicate that the A-domain is highly ‘prone’ to allow changes in effector recognition but at various unexpected secondary positions. More recently, this formed the basis to hypothesize that this domain readily adopts a *stem protein* configuration of ‘open’ flexibility towards new effector substrates [19].

In this work we extended the structural model of the XylR A-domain to predict that of the distantly related protein HbpR. Even though the basis for structural modeling of the A-domain is weak and only based on the alignment of the HbpR A-domain to the model of XylR A-domain, which on its turn is based on that of catechol O-demethylase (PDB entry 1vid), it allowed us to formulate a direct testable hypothesis for the implication of a number of amino acid residues in 2-HBP recognition. On the basis of the structural prediction and subsequent calculation of the energetically most favorable region for interaction of 2-HBP on the modeled protein surface by gramm [28], we identified one region with an exposed cavity (Fig. 2E, F). Indeed, site-directed mutagenesis of residues in this area identified several critical and non-critical amino acids for 2-HBP-mediated HbpR activation of the P_c_-promoter (Text S1). Notably, these were Glu184, emphasizing a residue with also critically conserved importance in XylR and DmpR (Text S1), Cys187 (conserved), and Ile180 (conserved), both of which had not been detected previously by mutagenesis on XylR or DmpR. Other residues, mutation of which reduced but not completely abolished activation potential by 2-HBP, were Ile56, Val182 and Leu207 (Text S1). HbpR-L207F seems to be produced less efficiently in *E. coli* (Fig. 4), but the other mutant HbpR proteins were were correctly produced in *E. coli*, albeit with detectable folding differences (Fig. 5). Hence, we conclude that these residues are of critical importance to 2-HBP effector mediation in HbpR. Since our *in vivo* assays only measure the outcome of 2-HBP-mediated activation by HbpR (mutants) on P_C_-expression, we cannot conclude whether those amino acid residues are implicated in 2-HBP ‘binding’ or in some other step of the activation pathway.

Highly speculatively, but still interesting, was the prediction of a cavity in the structural model of the HbpR A-domain surface where 2-HBP would interact. The importance of this cavity for 2-HBP mediated activation of HbpR was investigated further by systematically changing its residues (i.e., Val50, Thr52, Ile56, Leu60, Leu207 on one side, and Ile180, Val181, Val182, Glu183, Glu184, Ile185, Cys187, Gln188). Perhaps atypically, we chose to change residues in this region to the bulky phenylalanine with the prospect that such a bulky residue might physically block entry of 2-HBP to the cavity. However, some residues were changed multiple times, not only to phenylalanine but to less bulky residues, which essentially produced the same *in vivo* effects (Table 1, Text S1). Because bulky residues may change more than only the entry to the cavity we produced a number of control Phe-substitutions outside this region and analyzed HbpR mutant protein production in *E. coli*. Indeed seven substitutions severely impaired or abolished 2-HBP induction in *E. coli* (Ile56, Thr52, Ile180, Val182, Glu184, Cys187 and Leu207), whereas substitutions to residues positioned outside on the surface (Ser32, Glu42, Val50, Lys178) did not. Of all these, Ile180, Glu184 and Cys187 are conserved among XylR, DmpR and HbpR, whereas Thr52 is not (Text S1). Contrary to those critical residues, changes in Glu203, Glu183, Val181, Trp205 and Gln209 had no major effects, even though they were predicted to be near the cavity. It is likely that HbpR proteins with these mutations in their A-domains adopted slightly different local configurations than wild-type, as was demonstrated from circular dichroism scans of purified proteins (Fig. 5), but Western results did not indicate that this misfolded the protein completely (Fig. 4). Furthermore, certain Phe-substitution mutants with altered CD scans compared to wild-type HbpR did not display loss of function (Fig. 5, Table 1). Therefore, even though it is difficult to unequivocally decide whether bulky mutant residues cause loss of function because they change a critical residue or because they partial unfold the protein, our results suggests that this region is indeed a very critical one for proper 2-HBP-mediated activation of HbpR and that its most essential residues are Thr52, Ile180, Glu184 and Cys187. Interestingly, mutations in such residues did not change the effector spectrum of HbpR and neither did the other generated mutations, whereas previous mutations obtained by directed evolution of HbpR that did change the effector spectrum from 2-HBP to 2-chlorobiphenyl mapped in completely different areas of the A-domain (Text S1). Only some mutations, e.g. Ala202, mapped in a region which by directed evolution was shown to conceive a semi-constitutive phenotype (Text S1). This, and given the fact that three of them are conserved in XylR and DmpR (which do not react to 2-HBP), might suggest that the identified critical residues (e.g., Thr52, Ile180, Glu184 and Cys187) not so much directly ‘bind’ 2-HBP but are somehow important in transmitting an effector-interaction to activation of the transcriptional regulator. This for XylR and DmpR would consist of derepressing the ATPase activity, but for HbpR might consist of activating it [17].

Again, very suggestively but of potential importance for a novel activation mechanism concept, the observed predicted cavity enters into an opening the A-domain of HbpR (Fig. 1C). HbpR's A-domain is a little shorter at its C-terminal end and extends less into the B-linker than for XylR or DmpR, which makes this cavity more pronounced. Although the cavity is not visible in the XylR A-domain model as proposed by Devos [22], this is only because of the C-terminal extension of 12 residues in their model. Removal of the C-terminal beta-sheet shows that the XylR A-domain also adopts such a cavity and produces an opening (Fig. 1D). Obviously, this part of the modeling is highly speculative, because the A-domain part of the protein connects to the C-domain via the proposed flexible B-linker and this connection loop cannot be properly assigned a structure without a good template. Modeling of the C-domain of both HbpR and XylR is possible, because of reasonably high homology (41.2% for HbpR) to the resolved crystal structure of NtrC1 of *Aquifex aeolicus* (PDB entry 1NY5_B). Unfortunately, this homology does not extend into the B-linker region of some 30 amino acid residues (not shown), making structure predictions for the connecting region between A- and C-domains premature. The importance for mentioning this cavity, however, is that it conceptually would offer a new hypothesis for effector mediated activation of proteins in this family, which so far is not solved satisfactorily [31]. Instead of having a classical active site ‘pocket’, one could imagine that the flexible B-linker region occludes or ‘opens’ the proposed cavity and opening through which effectors pass, and that this triggers an intramolecular conformational change needed to expose the C-domain ATP-ase and activate RNA polymerase.

In conclusion, therefore, our results highlight the importance of a region on the HbpR A-domain for effector (2-HBP) control. Critical residues for effector control in this region were identified, some of which are conserved with XylR and DmpR, thus ruling out a direct role in effector binding. Model predictions were reasonably correct with experimental data. As a new hypothesis for effector mediated control on this type of proteins, we propose a model for activation in which the effector compound would pass through a surface crevice instead of binding to a pocket and subsequently being released from the same pocket. Whether or not the surface crevice needs to be made by a single protomer of the activator protein or a dimer remains to be determined. Direct binding studies of radio-actively labeled phenol to the DmpR A-domain indicated a fraction of up to 0.6 mol substrate bound per mol protein [32], which could be interpreted as a not yet saturated system in which one effector molecule would bind one protomer, or as a slightly oversaturated system in which one effector molecule binds a protomer dimer. Such a model for a surface crevice and tunnel would also help to understand the large variety of mutations that influence effector specificity and semi-constitutive phenotypes, which have been discovered over the years in XylR, DmpR and HbpR (Text S1). The reason for this would be that any mutation in the A-domain that somehow changes this crevice and permits entry to the tunnel may facilitate another effector to activate the complex. In addition, it could provide a mechanistic interpretation for the effector-mediated activation. The current hypothesis for activation states that only multimeric forms of XylR or HbpR (hexameric or heptameric, depending on the model) are capable of activating σ^54^ RNA polymerase. A plausible mechanistic model for such an activation process would be a slight conformational change induced in the hexameric complex by a torsion from a number of effector molecules passing through the cavity-opening.

## Materials and Methods

### Strains, media and general growth conditions

*E. coli* recombinant strains were generally cultivated on Luria Bertani (LB) medium [33], supplemented with 50 µg/ml kanamycin to select for the presence of the plasmid carrying *hbpR* or its mutants plus a transcriptional fusion between the HbpR regulatable P*_C_* promoter and *egfp*. For induction experiments *E. coli* strains were incubated in MOPS medium (i.e., per liter, 10 g 3-(*N-*morpholino)propanesulfonic acid, 1 g NH_4_Cl, 0.5 g NaCl, 0.06 g Na_2_HPO_4_·2H_2_O, 0.045 g KH_2_PO_4_, 20 mM MgCl_2_, 1 mM CaCl_2_, and 2 g glucose, pH 7). Liquid cultures were generally incubated at 37°C with 180 rpm rotary shaking, except for induction experiments, which were carried out at 30°C. Bacterial colonies were grown on LB medium solidified with 1.5% agar and incubated at 30°C or 37°C.

### HbpR activation assays

In order to test HbpR- or HbpR-mutant dependent *egfp* activation from the P*_C_* promoter in *E. coli* we applied the protocol essentially as described previously [30]. Single pure *E. coli* cultures were grown for 16 h at 37°C in LB medium plus kanamycin and diluted fifty fold in fresh medium of the same. Cells were regrown until the turbidity in the culture reached 0.4 (at 600 nm), after which they were centrifuged at 2,500× *g* and resuspended in the same volume of MOPS buffer. This cell suspension was used for induction assays. For induction, 200 µl of *E. coli* cell suspension was mixed with the effector (20 µM 2-HBP, 100 µM 2-chlorobiphenyl) in a 96-well microtitre plate. Plates were incubated at 30°C with 180 rpm rotary shaking for periods of 2 and 4 h, after which the EGFP fluorescence signal was measured per fluorimetry (FluoStar Galaxy, BMG Labtech GmbH, Offenburg, Germany). Incubations with dimethylsulfoxide and water only served as negative, non-induced controls. Assays were performed in triplicate. Induction experiments with 20 µM toluene were carried out in 2 ml glass vials closed by Teflon-lined caps to avoid evaporation. In this case 0.2 ml of the cell suspensions was transferred after 2 and 4 h into a 96-well microtitre plate for fluorescence measurements.

### DNA cloning techniques and DNA sequencing

Recombinant DNA techniques were all carried out according to well-established procedures [33]. PCR mutagenesis mixtures were prepared as suggested by the suppliers of the Pfu Polymerase (Promega) and run on GeneAmp PCR System thermocyclers (Applied Biosystems, CA, USA). DNA was sequenced using the BigDye Terminator cycling method (v3.1, Applied Biosystems) and analyzed on ABI Prism 3100 capillary sequencers (Applied Biosystems). Kits for purification of PCR products or of DNA fragments from agarose gels, and for isolation of plasmid DNAs from *E. coli* were used according to the specifications given by the suppliers (Qiagen, Promega).

### Chemical substances

2-hydroxybiphenyl (2-HBP), 2-chlorobiphenyl and toluene were obtained from Sigma-Aldrich. Stock solutions were prepared at 20 mM in dimethylsulfoxide, which were kept at 4°C in the dark. All other chemicals were of the highest purity grade available.

### Bioinformatics analyses

Common bioinformatics analyses were performed using tools provided by the ExPASy (**Ex**pert **P**rotein **A**nalysis **Sy**stem) proteomics server of the Swiss Institute of Bioinformatics (SIB) with default settings (http://swissmodel.expasy.org). The 218 first amino acids corresponding to the A-domain of the HbpR protein were structurally aligned using magic-fit to a pdb-model of those of XylR and DmpR (http://www.pdg.cnb.uam.es/XylR), and further refined with the help of the online Workspace program swiss-model [26], [27]. Further three-dimensional analyses were conducted using the DeepView/Swiss-Pdbviewer program version 4.01 for OS.X [26].

### Docking

Hypothetical docking positions of 2-HBP on HbpR were computed using the gramm program (Global Range Molecular Matching, [28]). The three-dimensional HbpR A-domain model and the 2-HBP pdb-files were submitted to gramm with the following parameters: Matching mode =  generic; grid step = 1.7; repulsion = 20.0; Attraction double range = 0.0; Potential range type = atom_radius; black white projection; representation = all; 1000 output matches, angle for rotation = 10.

### Site-directed *hbpR* mutagenesis

Site-directed HbpR mutants were constructed by PCR by using two different methods. In the first method, the *hbpR* gene was amplified in two parts independently by PCR, the junction of which carried the desired mutation. One fragment was amplified with the forward primer 040101 (A-domain For: 5′-gtcgacgcggccgcgcactttcgcacg-3′) and the reverse oligonucleotide carrying the desired mutation, whereas the second fragment was amplified using the reverse primer 040102 (A-domain Rev: 5′-tgcgcatgctcggaggatccggtttca-3′) and the forward complementary mutated oligonucleotide (Text S1). The two first PCR products were used as template for a second PCR reaction in which the two external primers 040101 and 040102 were used. PCRs fragments were separated on agarose gel and fragments of the proper size were purified by gel extraction (Qiagen). Purified PCR fragments were digested by *Sac*I and *Bam*HI and used to replace wild-type *hbpR* in the *egfp* expression plasmid pHBP269A0 to test the inducibility of the mutants for biaromatic compounds [30]. This plasmid carries *hbpR* under its own promoter and *egfp* under the control of the P*_C_* promoter. Moreover, the 5′ part of *hbpR* is modified to introduce 6 His-codons in the protein, after which the regular first Met-codon of HbpR is maintained. Plasmids were transformed into *E. coli* DH5α and re-extracted from transformants to confirm the introduced mutation by DNA sequencing. Six HbpR mutants (Ile180Thr, Glu183Gln, Glu184Leu, Ile185Thr, Glu203Leu and Trp205His) were constructed using this technique.

The other HbpR mutants were created by a method in which the whole plasmid was amplified by two reverse complementary primers carrying the mutations. For this purpose, we first cloned the *hbpR* A-domain gene region separately. The gene region with the A-domain was amplified by using the polymerase chain reaction and primers 040101 and 040102 (PCR cycle: 94°C for 2 min, followed by 25 cycles of each: 94°C for 2 min; 56°C for 30 sec; 72°C for 1 min). The PCR product was digested by *Sac*I and *Bam*HI and cloned into pUC18 digested by the same enzymes. pUC carrying the cloned A-domain gene fragment of *hbpR* was used as template for the mutagenic PCRs. These PCR was performed by using *Pfu* DNA polymerase mix and the following cycling regime: 94°C for 30 sec, followed by 16 or 18 cycles of each: 94°C for 30 sec; 55°C for 1 min; 68°C for 2 min.

The fully PCR-amplified plasmid was treated with *Dpn*I to remove parental (methylated) template plasmid and was then directly transformed into *E. coli* DH5α, during which the single-stranded breaks created by the PCR are repaired. Plasmids from potential transformants were purified and sequenced to confirm the mutation. In case of successful mutation, the A-domain gene regions were recovered by *Sac*I and *Bam*HI digestion, and used to replace the *hbpR* wild-type A-domain sequence on pHBP269A0. After transformation in *E. coli*, those plasmids were again purifed and verified for the integrity of the introduced mutation. If correct, the strains were used to test inducible *egfp* expression by 2-HBP from the HbpR controlled P*_C_* -promoter.

All *hbpR* mutant genes in pHBP269A0 subsequently transformed into an *E. coli* carrying the compatible plasmid pHBP124 with wild-type *hbpR* expressed from its native promoter [34], in order to test dominance of the created mutation.

### HbpR and HbpR mutant purification

HbpR and HbpR mutants were overexpressed and purified from *E. coli*. Hereto we fused the *hbpR* start codon to the ATG triplet present in the *Nde*I site of pET15d (Stratagene). This will produce an N-terminal His6-tag to *hbpR*. The *hbpR* gene was first amplified from the *P. azelaica* HBP1 chromosomal DNA by PCR with primers NdeI-HbpR (5′-GCCATATGAAATCAAATAAAAATAATAGCGAC- 3′; The *Nde*I site is underlined) and BamH1-HbpR (5′-GCGGATCCTATGTGATCTTTTTGACGCGGT-3′; the *Bam*HI site is underlined). The 1710-bp PCR product was digested by *Nde*I and *Bam*HI and ligated to pET15, digested with the same enzymes. After transformation into *E. coli* BL21 (DE3) this resulted in plasmid pHBP240. The integrity of the *hbpR* open reading frame was verified by DNA sequencing.

*E. coli* BL21(DE3) containing pHBP240 was grown at 30°C in LB medium to an optical density at 600 nm of 0.6. To induce T7 RNA polymerase expression isopropyl-β-D-thiogalactopyranoside was added at a concentration of 1 mM, and cultures were further incubated overnight at 20°C. Cells were then collected by centrifugation for 5 min at 12,000× *g*, washed in the same volume of buffer containing 20 mM Tris-HCl and 2 mM EDTA (pH 7), and again centrifuged. The bacterial pellet was resuspended in 1/10 volume of lysis buffer (50 mM NaH_2_PO_4_, 300 mM NaCl, 10 mM imidazole, 5 mM β-mercapto-ethanol, pH 7.5) containing 2,5 mM of Pefabloc SC (4-2-aminoethyl-benzenesulfonyl fluoride, Roche) as protease inhibitor, and subjected to ultrasonication for five times during 20 seconds each at 60% and 40 W output (Branson 450 Sonifier). The cell extract was centrifuged for 30 min at 8,000× *g* to remove cell debris. The supernatant (1/10 volume) was mixed with 1 ml of 50% Ni-NTA agarose (Qiagen) during 2 hours with stirring at 4°C, and the mixture was loaded on a polypropylene column (1 ml, Qiagen) equilibrated with 600 µl lysis buffer. After loading, the column was washed three times with 4 ml of washing buffer (2 mM EDTA, 300 mM NaCl, 5 mM β-mercapto-ethanol, pH 7.5) containing increasing amounts of imidazole (25 mM, 50 mM and 75 mM). His6-HbpR and any of the His6-HbpR mutants were eluted with elution buffer (50 mM NaH_2_PO_4_, 300 mM NaCl, 250 mM imidazole, 5 mM β mercapto-ethanol). Fractions of 0.5 ml were collected and analysed by conventional sodium dodecyl sulfate polyacrylamide gel electrophoresis. Fractions with the highest concentration of purified His6-HbpR protein were pooled and dialyzed overnight against dialysis buffer (10 mM Tris HCl, 20 mM KCl, 1 mM EDTA, 10% glycerol).

### Protein electrophoresis and immunoblotting

*E. coli* cultures expressing HbpR or mutant HbpR-s from pHBP269A0-configurations (see above) were cultured in 5 ml LB at 30°C to an OD of 0.5, after which cells were harvested by centrifugation from 1.5 ml. Cell pellets were resuspended in 50 µl of PBS solution, to which 50 µl of reducing 2xSDS sample buffer was added (120 mM Tris-HCl [pH 6.8], 2% [w/v] SDS, 10% [v/v] glycerol, 0.01% [w/v] bromophenol blue, 2% [v/v] 2-mercaptoethanol). Protein extracts were prepared by boiling the cell-loading buffer mixtures for 10 min. Appropriate volumes were loading on denaturing SDS-PAGE, containing 4% stacking and 8% separating gels (acrylamide∶bisacrylamide 29∶1; Bio-Rad), using the MiniProtean electrophoresis system (Bio-Rad) according to standard protocols [33], [35]. For immunoblotting, the proteins were transferred to polyvinylidene difluoride membranes (Immobilon, Millipore) using a semi-dry electrophoresis transfer apparatus (Bio-Rad) and then blocked in PBS buffer containing 3% skimmed milk and 0.1% Tween-20 for 1 h.

Because commercial anti-His-antibodies were not sufficiently sensitive to detect His_6_-HbpR expression in *E. coli* from the native P_R_-promoter, we used an M13-nanobody carrying antigen-binding part of camel antibodies (V_HH_) [36]. Purified HbpR was used to immunize an African camel and after a 6 week period, RNA was extracted from lymphocytes and retrotranscribed to DNA. Sequences corresponding to the antigen-binding domain were amplified and cloned in a phage display vector. Phage libraries were screened multiple times by phage-ELISA for the best binder, which was kept as a stock producer of the anti-HbpR M13-V_HH_ phagebody, as described in Zafra et al [36]. Membranes for Western were placed in phage suspensions diluted 1/1000 of the pool of M13 particles apically presenting V_HH_ domains for detection of HbpR protein in a PBS buffer supplemented with 3% skimmed milk, 0.1% Tween-20 and 0.1% sodium deoxycholate, followed by a 45 min incubation at room temperature with mild shaking. Unbound phages were washed out with four 5 min rinses with the same PBS/Tween-20/sodium deoxycholate buffer but devoid of milk. After washing with PBS, M13 capsids bound to the blotted proteins were detected by immersion of the membrane in a 1/5000 dilution of HRP/anti-M13 Monoclonal Conjugate and revealed with the BM chemiluminiscence Blotting Substrate-POD kit (Roche). Alter 1 min incubation in the dark, the blots were exposed to X-OMAT X-ray film (Kodak).

Gels were scanned and band intensities were corrected for the total intensity of protein bands on the corresponding SDS-PAGE Coomassie Blue stained gels, and then normalized for exposure differences by dividing by the mean band intensity. An average normalized intensity was then calculated on the basis of both the HbpR bands visible in Western. All average normalized intensities of HbpR mutants were then plotted in a Box-plot, to identify outliers below the 25% and above the 75% quantiles (Text S1).

### Circular dichroism

The CD spectrum of purified His6-HbpR or of its mutants was obtained from a J810 spectropolarimeter (Jasco, Tokyo, Japan) using a quartz cell with a 0.1-cm path length (*L*). CD spectra (θ, milli-degree) were measured at 25°C between 195 and 250 nm at a scanning speed of 10 nm/min and a protein concentration of ≈0.3 mg/ml. After subtracting the spectrum from background generated from buffer alone, the spectra for HbpR and its mutants were normalized to *delta epsilons* (Δε, mdegree·M^−1^·cm^−1^), using the protein concentration (*c*, mg·ml^−1^) and the mean residue weight of HbpR (MRW), via the formula (http://dichroweb.cryst.bbk.ac.uk/html/userguide.shtml):
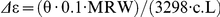



## Supporting Information

Text S1 Supplementary Materials.
(PDF)
