# Supplementary material for: Characterisation of the Putative Effector Interaction Site of the Regulatory HbpR Protein from Pseudomonas azelaica by Site-Directed Mutagenesis
Source: PLoS One. 2011 Feb 17;6(2):e16539. doi: 10.1371/journal.pone.0016539 (PMC3040749; doi:10.1371/journal.pone.0016539)
Supplement: Text S1 — Supplementary Materials. (PDF) [file pone.0016539.s001.pdf]

Supplementary Material to

Characterisation of the putative effector interaction site of the regulatory HbpR protein from *Pseudomonas azelaica* by site-directed mutagenesis.

Christelle Vogne[1], Hansi Bisht[2], Sagrario Arias[1], Sofia Fraile[3], Rup Lal[2], and Jan Roelof van der Meer[1*]

[1]Department of Fundamental Microbiology, University of Lausanne, 1015 Lausanne, Switzerland, [2]Department of Zoology, University of Delhi, India, [3]National Centre for Biotechnology-CSIC, Madrid 28049, Spain.

*Corresponding author:

J. R. van der Meer

Department of Fundamental Microbiology

University of Lausanne,

Bâtiment Biophore

Quartier UNIL-Sorge

1015 Lausanne, Switzerland

Tel. +41 21 692 5630

Fax.: +41 21 692 5605

Email: janroelof.vandermeer@unil.ch

Table S1. Nucleotide sequence of the primers used, with the introduced mutations shown in lower case.

Name	Sequence
S23F-For	5'- CTGCACTTTT**t**TCCCAACG -3'
V41F-For	5' -CCTACAA**t**T**t**GAGACACT G -3'
E42F-For	5' -CTGCTCCTACAAGTG**ttt**ACACTGAAGGATATAT -3'
T43F-For	5' -CCTACAAGTGGAG**ttt**CTGAAGGATATATAC -3'
I47T-For	5'- ACACTGAAGGATA**cc**TACAAGGAA -3'
L51N-For	5'- ATATACAAGGAA**aac**CAGGCCTATTCT -3'
K169F-For	5'- GCATGCGCTGGA**ttt**CCCATTGTCGTG G -3'
I171F-For	5'- GAAAACCC**t**TTGTCGTGG -3'
I171T-For	5'- TGCGCTGGAAAACCCA**cc**GTCG -3'
V172F-For	5'- GGAAAACCCATT**ttt**GTGGAAGAGATC G -3'
V172T-For	5'- GAAAACCCATT**ac**CGTGGAAGAG -3'
V173T-For	5'- AACCCATTGTC**acc**GAAGAGATCGA -3'
E174F-For	5'- AAAACCCATTGTCGTG**ttt**GAGATCGAATGCCAAG -3'
E174Q-For	5'- CCATTGTCGTG**c**AAGAGATCGAATG -3'
E175F-For	5'- CGTGGAA**ttt**ATCGAATG -3'
E175L-For	5'- CCATTGTCGTGGAA**ctc**ATCGAATGC -3'
I176T-For	5'- TCGTGGAAGAGA**c**CGAATGCCAAG -3'
C178F-For	5'- GGAAGAGATCGAAT**tt**CAAGCGATGGGAC -3'
Q179E-For	5'- ATCGAATGC**gag**GCGATGGGAC -3'
A193S-For	5'- TCAAGCGAAGCCC**ag**CGAAATGTGG -3'
E194L-For	5'- AAGCCCGCC**ctc**ATGTGGGCGCTC -3'
E194P-For	5'- CAAGCGAAGCCCGCC**ccc**ATGTGG -3'
E194Q-For	5'- AAGCCCGCC**c**AAATGTGGGCG -3'
W196H-For	5'- GCCGAAATG**cac**GCGCTCAGTCAG -3'
L198F-For	5'- CGCCGAAATGTGGGCG**t**T**t**AGTCAGTCGGAGCAAT -3'
Q200L-For	5'- GCGCTCAGT**ttt**TCGGAG -3'
Q200F-For	5'- GGCGCTCAGTC**tc**TCGGAGCAA -3'

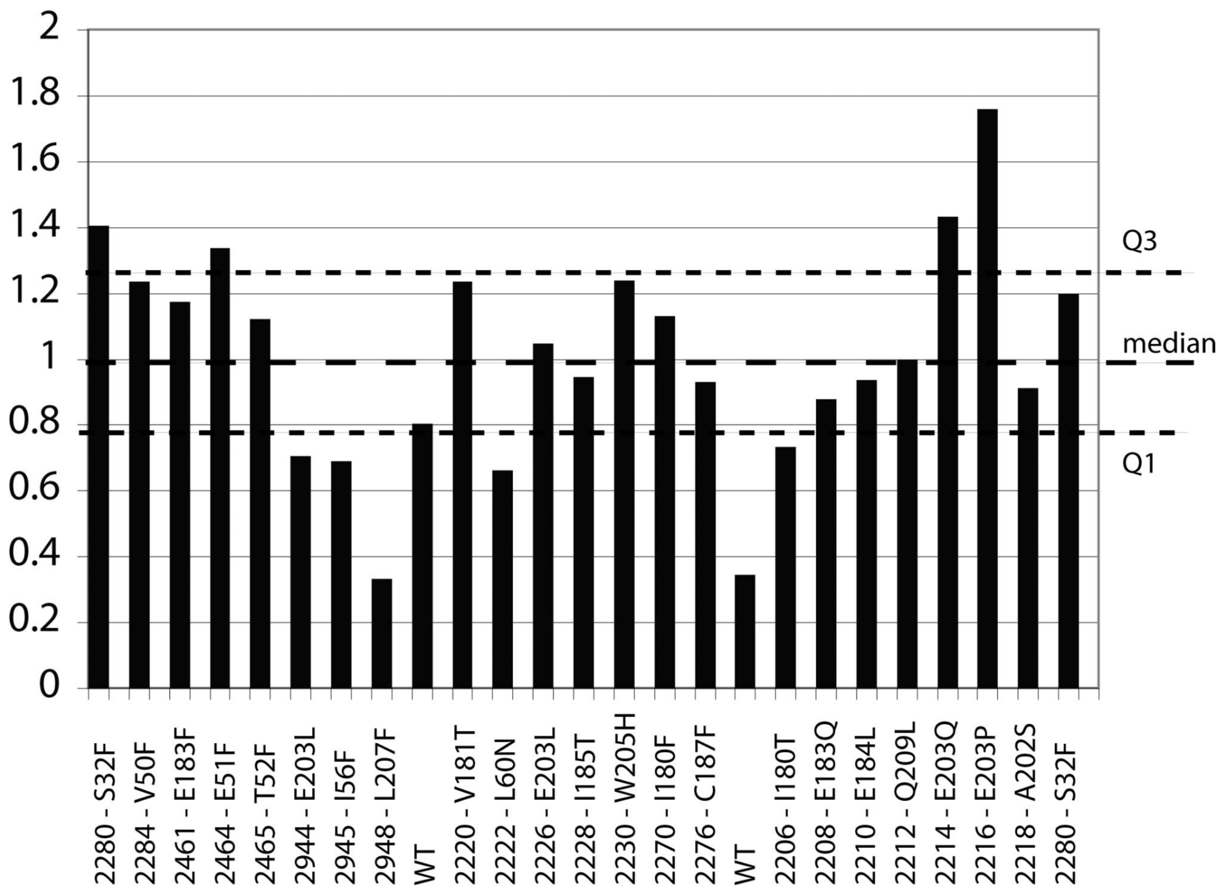

Fig. S1. Normalized band intensities of HbpR proteins expressed from the P_R-promoter in *E. coli* as detected by the anti-HbpR M13-V_{HH} phage antibody. Protein band intensities on Western (Fig. 4) were normalized for film exposure differences and for the total amount of protein loaded, and then averaged over both HbpR bands. This average intensity is plotted in the graph, with the calculated median, the 25% quantile (Q1) and the 75% quantile (Q3) over all cultures. HbpR expression in strains 2948 (L207F), 2216 (E203P) and one of the wild-type are considered outliers in the box plot calculation.

```
gi|455334|gb|AAB59162.1|       --------MSLTYKPKMQHEDMQDLSSQIRFVAAEGKIWLGEQRMLVMQL 42
gi|483552|emb|CAA48174.1|      --------MPIKYKPEIQHSDFKDLTNLIHFQSMEGKIWLGEQRMLLLQF 42
                                                                                L
gi|2098614|gb|AAB57638.1|      MKSNKNNSDDRSIVADLALPEVHALVSKLHFSPNEGRIWLDESRCLLLQV 50
gi|1633081|pdb|1VID|           --------MGDTKEQRILRYVQQNAKPGDPQSVLEAIDTYCTQKEWAMNV 42


gi|455334|gb|AAB59162.1|       STLASFRREIISLIGVERAKGFFLRLGYQSGLMDAELARKLRPAMREEEV 92
                                                     L                   S
gi|483552|emb|CAA48174.1|      SAMASFRREMVNTLGIERAKGLFLRHGYQSGLKDAELARKLRPNASEVGM 92
                                                             D         P ?
gi|2098614|gb|AAB57638.1|      ETLKDIYKELQAYSGPDYTREFLTRIGFTTGQRDAEMIIKKQGISSIKEQ 100
gi|1633081|pdb|1VID|           G---DAKGQIMDAVIREYSPSLVLELGAYCGYSAVRMARLLQPGARLLTM 89


gi|455334|gb|AAB59162.1|       FLAGPQLYALKGMVKVRLL----TMDIAIRDGRFNVEAEWIDSFEVDICR 138
                                                                  A          E/N/Q
gi|483552|emb|CAA48174.1|      FLAGPQMHSLKGLVKVRPT----ELDIDKEYGRFYAEMEWIDSFEVEICQ 138
                               L               C       V  N     L P       LK A/D/K/R
gi|2098614|gb|AAB57638.1|      IYAGGVLHALQGFLTSIQAGSSALNAVDMKSMDYHAEAYWQNSIEAEIHL 150
gi|1633081|pdb|1VID|           EMNPDYAAITQQMLNFAGLQ---DKVTILNGASQDLIPQLKKKYDVDTLD 136


gi|455334|gb|AAB59162.1|       TELGLMNEPVCWTVLGYASGYGSAFMGRRIIFQETSCRGCGDDKCLIVGK 188
                                                                K           I
gi|483552|emb|CAA48174.1|      TDLGQMQDPVCWTLLGYACAYSSAFMGREIIFKEVSCRGCGGDKCRVIGK 188
                                K                                    ?      W   E
gi|2098614|gb|AAB57638.1|      AMHGVSSHAVCWFSVAYCSGYLSACAGKPIVVEEIECQAMGHTHCRIQAK 200
gi|1633081|pdb|1VID|           MVFLDHWKDRYLPDTLLLEKCGLLRKGTVLLADNVIVPGTPDFLAYVRGS 186


gi|455334|gb|AAB59162.1|       TAEEWGDVSSFEAYFKSDPIVD-------------- 210
gi|483552|emb|CAA48174.1|      PAEEWDDVASFKQYFKNDPIIE-------------- 210
                                   R
gi|2098614|gb|AAB57638.1|      PAEMWALSQS-------------------------- 210
gi|1633081|pdb|1VID|           SSFECTHYSSYLEYMKVVDGLEKAIYQGPSSPDKS 221
```

Fig. S2. CLUSTAL 2.0.10 multiple sequence alignment of XylR (AAB59162), DmpR (CAA48174) and HbpR (AAB57638) A-domains compared to the catechol O-methylase (1VID). Major residual mutations in XylR and DmpR are indicated below the corresponding amino acid. For an HbpR A-domain mutation compilation, see Fig. S2. Mutants and mutant effects described in: [1] [2] [3] [4] [5] [6] [7] [8].

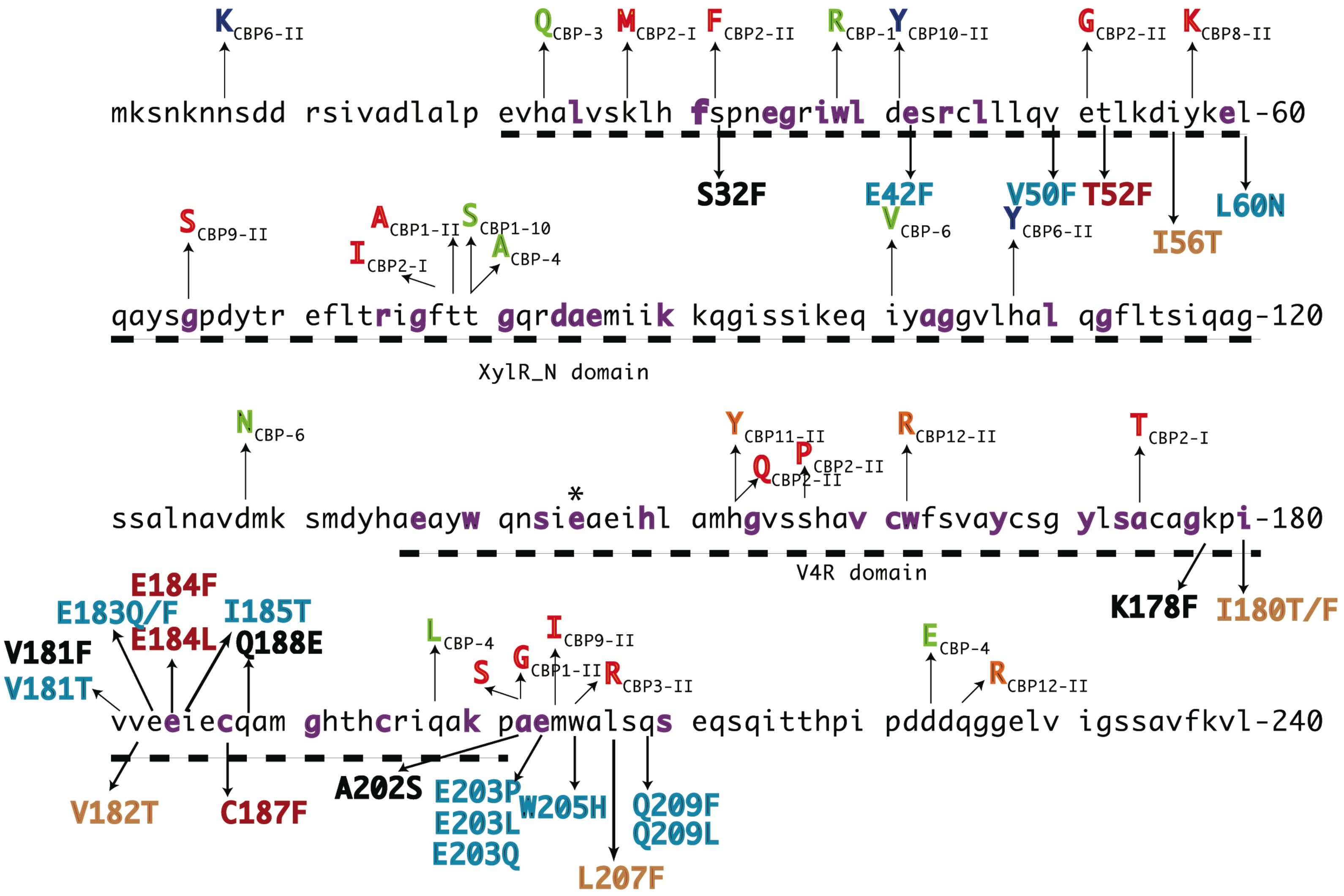

mksnknnsdd rsivadlalp evhalvsklh fspnegriwl desrclllqv etlkdiykel-60
K CBP6-II
Q CBP-3
M CBP2-I
F CBP2-II
R CBP-1
Y CBP10-II
G CBP2-II
K CBP8-II
S32F
E42F
V50F
T52F
L60N
I56T
qaysgpdytr efltrigftt gqrdaemiik kqgissikeq iyaggvlhal qgfltsiqag-120
S CBP9-II
A CBP1-II
S CBP1-10
I CBP2-I
A CBP-4
V CBP-6
Y CBP6-II
XylR_N domain
ssalnavdmk smdyhaeayw qnsieaeihl amhgvsshav cwfsvaycsg ylsacagkpi-180
N CBP-6
Y CBP11-II
Q CBP2-II
P CBP2-II
R CBP12-II
T CBP2-I
V4R domain
K178F
I180T/F
vveeiecqam ghthcriqak paemwalsqs eqsqitthpi pdddqggelv igssavfkvl-240
E184F
E183Q/F
I185T
E184L
Q188E
V181F
V181T
L CBP-4
I CBP9-II
G CBP1-II
S
R CBP3-II
E CBP-4
R CBP12-II
V182T
C187F
A202S
E203P
E203L
E203Q
W205H
Q209F
Q209L
L207F

Fig. S3. Primary sequence of the HbpR A-domain and the position and changes of site-directed and randomly selected mutations. Residues in pink are identical to those in aligned XylR and DmpR A-domains. Dotted lines below the primary sequence point to the two predicted conserved XylR_N and V4R domains. Residues indicated as e.g., S32F, are those produced in this study, with red labeled residues abolishing 2-HBP induction, orange diminishing induction, blue having no major effect, and black demonstrating higher background expression in absence of 2-HBP, as compared to wild-type. Residues labeled e.g., $S_{CBP9\text{-}II}$, point to those recovered from directed evolution experiments in a previous study [9], with green meaning gain of induction potential with 2-chlorobiphenyl, blue indicating gain of function with elevated background; orange, elevated background but no gain of function nor loss of 2-HBP inducibility; and red, loss of inducibility but semi-constitutive phenotype.

References

1. Sarand I, Skarfstad E, Forsman M, Romantschuk M, Shingler V (2001) Role of the DmpR-mediated regulatory circuit in bacterial biodegradation properties in methylphenol-amended soils. Appl Environ Microbiol 67: 162-171.

2. Galvao TC, Mencia M, de Lorenzo V (2007) Emergence of novel functions in transcriptional regulators by regression to stem protein types. Mol Microbiol 65: 907-919.

3. Garmendia J, Devos D, Valencia A, de Lorenzo V (2001) A la carte transcriptional regulators: unlocking responses of the prokaryotic enhancer-binding protein XylR to non-natural effectors. Mol Microbiol 42: 47-59.

4. Shingler V, Pavel H (1995) Direct regulation of the ATPase activity of the transcriptional activator DmpR by aromatic compounds. Mol Microbiol 17: 505-513.

5. Delgado A, J.-L. R (1994) Genetic evidence for activation of the positive transcriptional regulator XylR, a member of the NtrC family of regulators, by effector binding. J Biol Chem 269: 8059-8062.

6. Salto R, Delgado A, Michan C, Marques S, Ramos JL (1998) Modulation of the function of the signal receptor domain of XylR, a member of a family of prokaryotic enhancer-like positive regulators. J Bacteriol 180: 600-604.

7. Skarfstad E, O'Neill E, Garmendia J, Shingler V (2000) Identification of an effector specificity subregion within the aromatic-responsive regulators DmpR and XylR by DNA shuffling. J Bacteriol 182: 3008-3016.

8. Pavel H, Forsman M, Shingler V (1994) An aromatic effector specificity mutant of the transcriptional regulator DmpR overcomes the growth constraints of Pseudomonas sp. strain CF600 on para-substituted methylphenols. J Bacteriol 176: 7550-7557.

9. Beggah S, Vogne C, Zenaro E, van der Meer JR (2008) Mutant transcription activator isolation via green fluorescent protein based flow cytometry and cell sorting. Microb Biotechnol 1: 68-78.
